# Vitamin D3 improves the effects of low dose Der p 2 allergoid treatment in Der p 2 sensitized BALB/c mice

**DOI:** 10.1186/s12948-016-0044-1

**Published:** 2016-08-05

**Authors:** Claudia Petrarca, Emanuela Clemente, Valentina Amato, Alessia Gatta, Sara Cortese, Alessia Lamolinara, Cosmo Rossi, Stefania Zanotta, Gianni Mistrello, Roberto Paganelli, Mario Di Gioacchino

**Affiliations:** 1 Unit of Allergy and Immunotoxicology, Center of Ageing Science, “Università G. d’Annunzio” Foundation, Chieti, Italy; 2 Department of Medicine and Ageing Science (DMSI), University “G. d’Annunzio” of Chieti-Pescara, Chieti, Italy; 3 Unit of Immuno-oncology, Center of Ageing Science, “Università G. d’Annunzio” Foundation, Chieti, Italy; 4 Animal Facility, Center of Ageing Science, “Università G. d’Annunzio” Foundation, Chieti, Italy; 5 Research Center, Lofarma SpA, Milan, Italy

**Keywords:** Allergen immunotherapy, Vitamin D3, Der p 2, Allergoid, Asthma, Allergy, T regulatory cells, Eosinophils, IgE, IL-10

## Abstract

**Background:**

Airborne allergens can induce an immunological chronic disease characterized by airway hyper responsiveness and inflammation, mediated by exaggerated Th2 immune response. Allergen-specific immunotherapy (AIT) is effective for treating this condition because it is able to modify its natural course by opposing the underlying pathogenic mechanisms and determining immune suppression, immune deviation and tolerance. The rational for the present study was to investigate the possibility of improving allergoid-based IT in terms of efficacy and safety. Recently, 1α,25-dihydroxyvitamin D_3_ (VD_3_), the active metabolite of vitamin D_3_, was described to be a potent inducer of T regulatory cells and to be a good adjuvant in AIT settings.

**Methods:**

We investigated whether the co-administration of VD_3_ could potentiate the effect of AIT even when added to a low dose of chemically-modified monomeric allergoid of Der p 2 (d2-OID), in a Derp p 2 (d2)-sensitized BALB/c mice model. Control groups where treated with sham, VD_3_ alone or d2-OID only.

**Results:**

The d2-OID alone was not fully successful, as expected for a low dose. VD_3_ administration was associated with some valuable, although limited, changes in the immunological parameters in the lung. On the contrary, the VD_3_ adjuvated allergoid vaccine induced the most prominent reduction of airway eosinophilia and Th2 cytokines and concomitant increase of T regulatory cells and IL-10 in the lung and Der p 2-specific IgG2a in the serum.

**Conclusions:**

The addition of VD_3_ to a conventional AIT protocol would allow the reduction of allergoid dose needed and therefore, the production costs. Moreover, beneficial immunomodulatory effects have been achieved by the oral administration which might favour the management of the therapy by the patients and their adherence, possibly enhancing the efficacy of the treatment.

**Electronic supplementary material:**

The online version of this article (doi:10.1186/s12948-016-0044-1) contains supplementary material, which is available to authorized users.

## Background

One of the most reliable mechanisms explaining the desensitizing action of AIT (allergen immunotherapy) is based on its ability to induce tolerogenic dendritic cells (DC) characterized by an immature immunophenotype (iDC, DC_0_); these latter are crucial in favouring the differentiation of adaptive CD4^+^CD25^+^Fopx3^+^ T regulatory cells (Tregs) stemming from peripheral T-cells [1]. The Tregs have the property of inducing anergy of allergen-specific Th2 effector cells through the secreted inhibitory cytokine IL-10 [2]. In clinical settings, effective AIT has been also associated with increased production of allergen-specific IgG4 antibodies [3]: these are thought to play a protective role by acting as blocking antibodies (which compete with the allergen-specific IgE, thus preventing the degranulation of effector cells) and by hampering the IgE-mediated presentation of the allergen to T cells [4]. Likewise, successful AIT was associated with an increase of IgG2a antibodies in mouse models of Der p 2 -induced allergic asthma [5, 6], although such beneficial role of IgG is not generally proved in other mouse models.

Over the years, several scientific efforts aimed at enhancing the efficacy and safety of AIT. The biologically active form of vitamin D_3_ (VD_3_) seems a good adjuvant for AIT as for its ability of modulating the innate and adaptive immune response [7–9] and inhibit DCs differentiation maintaining them in a persistent state of immaturity, through the down-regulation of co-stimulatory molecules and reduction of pro-inflammatory cytokines [10]; thus making them unable to activate alloreactive T cells [7]. VD_3_ is able to restore IL-10 secretion by Treg cells isolated from steroid-resistant asthmatics [11] and has the capacity to expand functional Tregs, able to suppress the Th2-driven response upon transfer in OVA-sensitized asthmatic mice [12]. Furthermore, VD_3_ is able to expand in vitro PBMC-derived human Tregs (Foxp3^hi^) obtained from atopic allergic subjects [13].

The concept of using VD_3_ as a down modulator of allergic diseases has been further explored in animal models. In OVA sensitized mice, the co-administration of VD_3_ plus OVA significantly inhibited the airway hyper-responsiveness and potentiated the AIT effect by further reducing serum OVA-specific IgE level, airway eosinophilia and Th2-related cytokines; the boosting effect of VD_3_ seems to be mediated by IL-10 and TGF-β, since the levels of these cytokines were elevated in the VD_3_-treated mice and the beneficial effect was abrogated in the presence of antibodies directed to these cytokines [14]. Moreover, VD_3_ supplementation confers durability of the beneficial effects of OVA-specific IT in VD_3_-deficient OVA-sensitized mice [15], suggesting the instauration of an effective tolerance.

In another study, VD_3_ was covalently linked to the major cat allergen Fel d 1 to be administered as desensitizing treatment to allergic mice. The VD_3_-Fel d 1 vaccine, as well as the conventional one, induced serum allergen-specific IgE-to-IgG isotype switch and reduction of Th2 cytokines; interestingly, the VD_3_-containing vaccine was more potent in inhibiting the allergen-induced airway symptoms, especially the eosinophilic inflammation [16]. The capacity to hold back the recruitment of eosinophils, possibly linked to the reduction of IL-5 levels, was observed also in another experimental setting [17]. VD_3_ has been tested in clinical settings too: in asthmatic children, VD_3_ administration significantly potentiates the AIT (subcutaneous) outcome, still promoting the increase of Treg frequency and immunosuppressive IL-10 and TGF-β cytokines expression [18]; while in another study, in children with allergic rhinitis, VD_3_ supplementation combined with AIT (sublingual) was more effective in the reduction of nasal and asthma symptoms [19]. Interestingly enough, VD_3_ has been proposed to be beneficial also in the setting of autoimmune disease patients affected by lupus through the decrease of the Th1-type immune response and Th1/Th2 ratio and increase in Treg [20]. Since VD_3_ can improve the effects of AIT, as widely demonstrated by the mentioned studies, its efficacy may be achieved also by reducing the vaccine doses. Such decrease of the amount of allergen in human vaccines would lower the cost of the therapy, considered an essential element for adherence. For this reason, the major purpose of the present study is to verify if a low dose of AIT associated to VD_3_ maintains similar effects to those of common AIT at usual doses, in the BALB/c mouse model of type I allergy towards Der p 2.

## Methods

### Animals

Specific pathogen free BALB/c mice (female, 8 weeks-old, 20–25 g of weight) were purchased from Charles River (Milan, Italy). The animals were housed in plastic cages with absorbent bedding material and amusement tools and were maintained on a 12 h daylight cycle. During the experiment, the mice were fed with “Altromin R” containing 2.0 IU of VD_3_ per gram of food and 9.8 g/kg of calcium. Food and water were provided ad libitum.

### Sensitization, challenge, and AIT protocol

Briefly, mice were sensitized with i.p. injections of 1 µg recombinant d2 adsorbed onto 2 mg Al(OH)_3_ (Lofarma, Milan, IT) in a total volume of 100 µl of pyrogen-free saline on days 0 and 14. Al(OH)_3_ served as an adjuvant to favor a Th2 response towards. The mice were challenged through i.p. injections with 1 µg recombinant d2 on day 37, and with aerosolised 1 % HDM extract in pyrogen-free saline to induce airway inflammation during and after the AIT period (days 27, 44, 60, 62, 64). The allergoid of the Der p 2 antigen was used as the vaccine tool for AIT, characterized by reduced availability of IgE binding sites while preserving epitopes necessary for T cell recognition and for induction of non pathogenic IgG blocking antibodies [21–24].

The sensitized and challenged mice received 3 µg of d2-OID alone (group d2-OID) or in combination with 120 ng of VD_3_ (group d2-OID + VD_3_). The production and purification regarding all forms of allergen/allergoid and the assessment of the low dose to be used in AIT—i.e. 3 µg—are fully described in the Additional file 1.

As controls, one group of sensitized mice was treated with 120 ng (group VD_3_) of VD_3_ alone, or saline (group Sham) following the same time schedule. The dose of VD_3_ was chosen on the basis of literature data [17, 25]. For these experiments, the biologically active form of VD_3_, 1,25(OH)_2_VD_3_ (Sigma Aldrich, Milan, IT) was dissolved in 96 % ethanol and freshly diluted in d2-OID solution. Then, 48 h after the last aerosol challenge, blood, bronchoalveolar lavage fluid (BALF), lung and spleen were harvested further analysis. The type of mice treatments and the time schedule were performed as described in the Additional file 1.

### Determination of serum levels of d2-specific antibodies

After 48 h from the last HDM aerosol challenge, blood was drawn from each mice via cardiac puncture. After blood coagulation had occurred, sera were stored at −20 °C until levels of d2-specific and total IgE and IgG2a were determined by enzyme-linked immunosorbed assay (ELISA). In brief, for d2-specific IgE titration, 96-wells microtiter plates (Maxisorp, Nunc) were coated with 100 µl of recombinant d2 at 1 µg ml^−1^ in 0.1 M NaHCO_3_ buffer (pH 9.5). After overnight incubation at 4 °C, the plates were washed and blocked with 2 % bovine serum albumin in PBS for 2 h at room temperature (RT). Diluted serum samples (1:100 for IgE and 1:500 for IgG2a) were incubated with the plate for 2 h, at RT. Next, after multiple washings, the wells were incubated for 2 h at RT with goat anti-mouse IgE isotype or IgG2a isotype (Bethyl laboratories, Montgomery, USA) diluited in 0.1 % bovine serum albumin (BSA) in PBS buffer (1: 1000 for IgE and 1:1000 for IgG2a). Rabbit anti-goat Ig-peroxidase (Bethyl) diluited in 0.1 % BSA in PBS buffer (1:10,000) was added for 1 h at RT. For color development, the chromogenic substrate TMB (Sigma-Aldrich) was added and, after an incubation of 15 min, the reaction stopped by adding 1 N H_2_SO_4_. The absorbance was measured at 450 nm using the Lambda 650 UV/Vis spectrophotometer (Perkin Elmer, Milan, Italy). Each sample was assessed in duplicate wells, in two experiments. The concentration of total IgE in each sample was calculated by extrapolation against the standard curve obtained by measuring samples of known concentration. The antibody levels (mean ± S.D.) were expressed in ng ml^−1^ (total Ig) or in optical density units (anti-d2 Ig).

### Preparation of BALF and lung tissue homogenate

Post sacrifice, BALF was collected by gentle injection of ice-cold PBS (0.3 ml) into the trachea, three times. The fluid was centrifuged (1200*g* × 5 min at 4 °C) and the supernatant was harvested at −80 °C for further analysis of the cytokines level by ELISA.

Also tissue homogenate was prepared from one lobe of lung tissue to be used to measure cytokine levels. The lung tissue was placed into a microcentrifuge tube, snap-frozen in liquid nitrogen and stored at −80 °C until further analysis. The frozen lungs were thawed, transferred to different tubes on ice containing 1 ml of Protease Inhibitor Cocktail (Sigma Aldrich, Milan, Italy). The lung tissues were homogenized at 4 °C with gentle MACS Dissociator Miltenyi Biotec, Bologna, Italy). Lung homogenates were centrifuged at 9000×*g* for 10 min at 4 °C. Supernatants were transferred to clean microcentrifuge tubes, frozen on dry ice for storage and thawed on ice for analysis. Total protein concentrations in the lung tissue homogenates were determined using a BCA kit (Sigma Aldrich). Lung tissue homogenates were diluted with buffer to a final protein concentration of 500 μg ml^−1^.

### Cytokine levels determination in BALF and in lung tissue homogenate

Cytokines, namely IL-1β, IL-2, IL-4, IL-6, IL-10, INF-γ and TNF-α, were analyzed in BALF samples and lung tissue homogenate supernatants by multiplex ELISA assay based on a fluorimetric method (Searchlight, Aushon Biosystem, MA, USA). Each sample (50 µl out of 1 ml for BALF or 500 µg ml^−1^ total protein for homogenate) was assessed in duplicate and the concentration of each cytokine was calculated by extrapolation against the standard curves obtained by measuring cytokine samples of known concentration using the Cirasoft™ Analyst software (Aushon Biosystem). The level of IL-13 was determined by colorimetric ELISA using a commercial kit (Peprotech, DBA, Milan, Italy) according to the manufacturer’s instructions. Data are expressed as referred to 1 ml of BALF or 500 µg total protein homogenate.

### Histochemical analysis of the lung

The lungs were inflated and fixed with 10 % buffered formalin after collection of BALF. Sample were embedded in paraffin and then sectioned. To ensure systematic uniform and random sampling, lungs were cut transversally, to the trachea, into 2.0 mm thick parallel slabs with a random position of the first cut in first 2 mm of the lung, resulting in 5–8 slabs for lungs. The slabs were then embedded cut surface down and sections were stained with hematoxylin and eosin (BioOptica, Milan, Italy) for detecting inflammatory cell infiltrates. Briefly, images of three random sections within the left lung proximal to the main stem bronchus were acquired under the optical microscope Upright Nikon Microphot SA) at 200× and 400× magnification, photographed with the Nikon DXM 120 color camera (Nikon Instruments, Melville, NY) and analyzed with the Act-1 software.

### Evaluation of T regulatory cells frequency in spleen

Single cells suspensions of spleens were prepared by squeezing through 70 µm strainers (BD Labware) and, after erythrocytes osmotic lysis with Hybri-MaxTM (Sigma-Aldrich), were stained for flow cytometry analysis using LIVE/DEAD^®^ Fixable Aqua Stain (Thermofisher Scientific, Milan, Italy), anti-CD4-FITC, anti-CD25-APC and anti-FoxP3-PE of the Mouse Treg detection kit (Miltenyi Biotec), according to manufacturer instruction. FACS analyses were performed using a FACSCanto II and the data analyzed using FACSDiva Software 6.0 (BD Biosciences). Single-stained and “fluorescence minus one” (FMO) samples were used as a compensation and analysis controls. The following gating hierarchy was applied: cell debris were excluded by setting a first gate in a FL-2 vs FL-3 (mock channel) dot plot; second, a forward light scatter/side light scatter (FSC/SSC) gate was applied on lympho-monocytes; third, a gate on CD4^+^ T cells was set and the percentage of Foxp3^+^CD25^+^ T cells within this population was determined. For each sample, twenty-thousand events were acquired within the lymphocytes gate. Experiments were performed in duplicate.

### Immunohistochemical detection of FoxP3^+^ cells and CD3^+^ cells in the lung

For immunoistochemistry, slides were deparaffinized and incubated with the following primary antibodies: CD3 (AB828, Rabbit anti-Human, Abcam, Milan, Italy) or FoxP3 (14 5773-82, Rat anti-Mouse, eBioscience, CA, USA) followed by the appropriate biotinylated secondary antibody (Jackson ImmunoResearch Laboratories, Milan, Italy). Immunoreactive antigens were detected using Streptavidin Peroxidase (Thermo Scientific-Lab Vision Corporation, CA, USA) and DAB Chromogen System (Dako Corporation, CA, USA) or Vulcan Fast Red Chromogen System (Biocare Medical, CA-USA). After chromogen incubation, slides were counterstained in Hematoxylin (BioOptica, Milan, Italy) and images were acquired by Leica DMRD optical microscope (Leica, United Kingdom). The rate of Treg cells was measured using a semiquantitative method and expressed as the number of FoxP3^+^ cell counts divided by the CD3^+^ cells scoring in five visual fields from different areas (sections): 1 was attributed to samples with null or rare CD3^+^ cells, score 2 corresponded to some positive cells and score 3 was given to samples with numerous positive cells.

### Differential cell counts in the BALF

BALF was performed by gentle injection of ice-cold PBS (0.3 ml) into the trachea, three times. The collected fluids were centrifuged (1200*g* × 5 min at 4 °C) and the cellular pellet was recovered for differential cell count. The cells were immobilized onto glass slides by cytospin preparation and, after air dry fixation, were stained with hematoxylin and eosin (both from BioOptica, Milan, Italy). A total of 300 total cells were counted in each slide using a hematocytometer. The percentage of macrophages, lymphocytes/mononuclear cells, eosinophils and neutrophils was determined by microscopic observation according to standard morphological criteria under 400× magnification.

### Statistical analysis

Values are expressed as mean ± SD. GraphPad Software 6.0 (Prism) was used for statistical analysis. Unpaired Student t test was used to determine differences between two groups. One-way analysis of variance (ANOVA) with Bonferroni correction was used for multiple groups comparison. The p value less than 0.05 was considered statistically significant (*p < 0.05, **p < 0.01, ***p < 0.001).

## Results

### Immunoglobulin levels in the serum

**Fig. 1:**
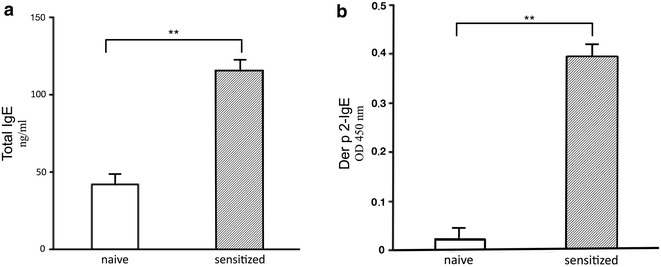
Evaluation of the allergic sensitization state in mice. Detection of total and anti-d2 specific serum IgE to evaluate the occurrence of allergic sensitization in mice. The mice were sensitized by 2 i.p. with 1 µg of recombinant d2, and the level of IgE in serum was measured 1 week after the last i.p. Significantly higher total IgE (**a**) and d2-specific IgE (**b**) levels are found in serum of sensitized mice compared with naïve mice. Values are expressed as mean ± SD (n = 4). **p < 0.01 compared with naive mice

After sensitization, IgE levels were significantly higher in the group of mice only sensitized and not treated compared with the naïve group (not sensitized, not treated): either total (+64 %, p < 0.01) (Fig. 1a) and anti-d2 specific IgE (+95 %, p < 0.01) (Fig. 1b).

**Fig. 2:**
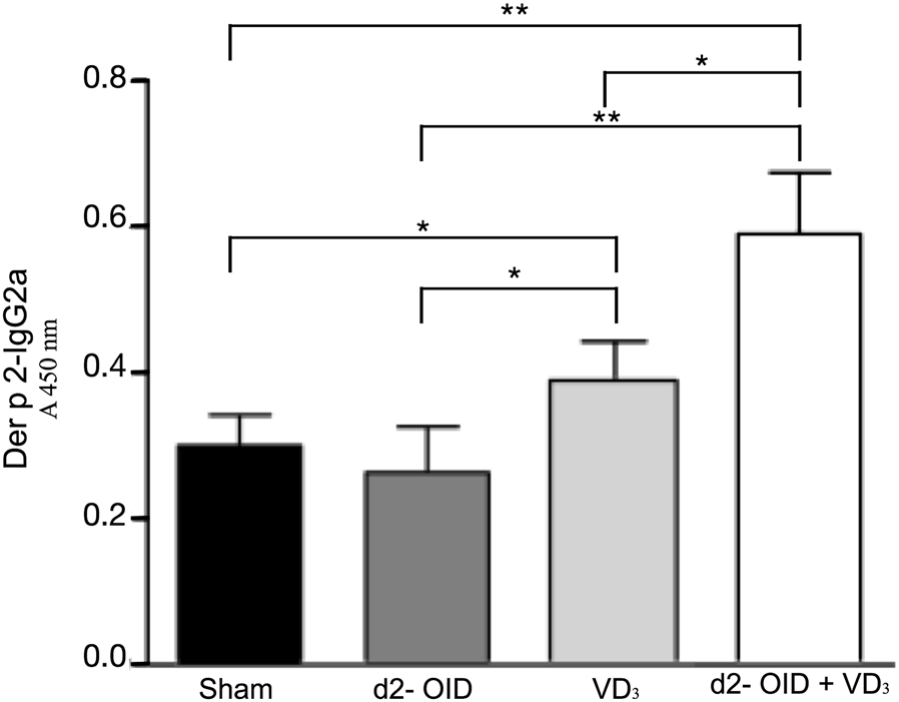
Serum levels of d2-specific antibodies. Specific antibodies levels in serum were measured by ELISA 48 h after the last HDM aerosol challenge, to determine the effect of different treatments on the humoral response. d2-specific IgG2a level was most significantly increased in the d2-OID + VD_3_ group compared to sham- and d2-OID-treated mice (p < 0.01). Values are expressed as mean ± SD (n = 12). *p < 0.05; **p < 0.01

Decrease of anti-d2-IgE was observed in all treated mice compared to sham group (not shown). Instead, only d2-OID + VD_3_ treatment induced the most significant increase of d2-specific IgG2a levels; more precisely, IgG2a level for this group was significantly higher than that measured in the sham- and allergoid-treated groups (+55 %, p < 0.01 vs both) and for the VD3-treated group (p < 0.05) (Fig. 2).

### Cytokine levels in lung homogenate and BAL fluid

Similar modifications were measured in the BAL fluid and lung homogenate for all the cytokines tested and only those relative to the first are described as follow.

**Fig. 3:**
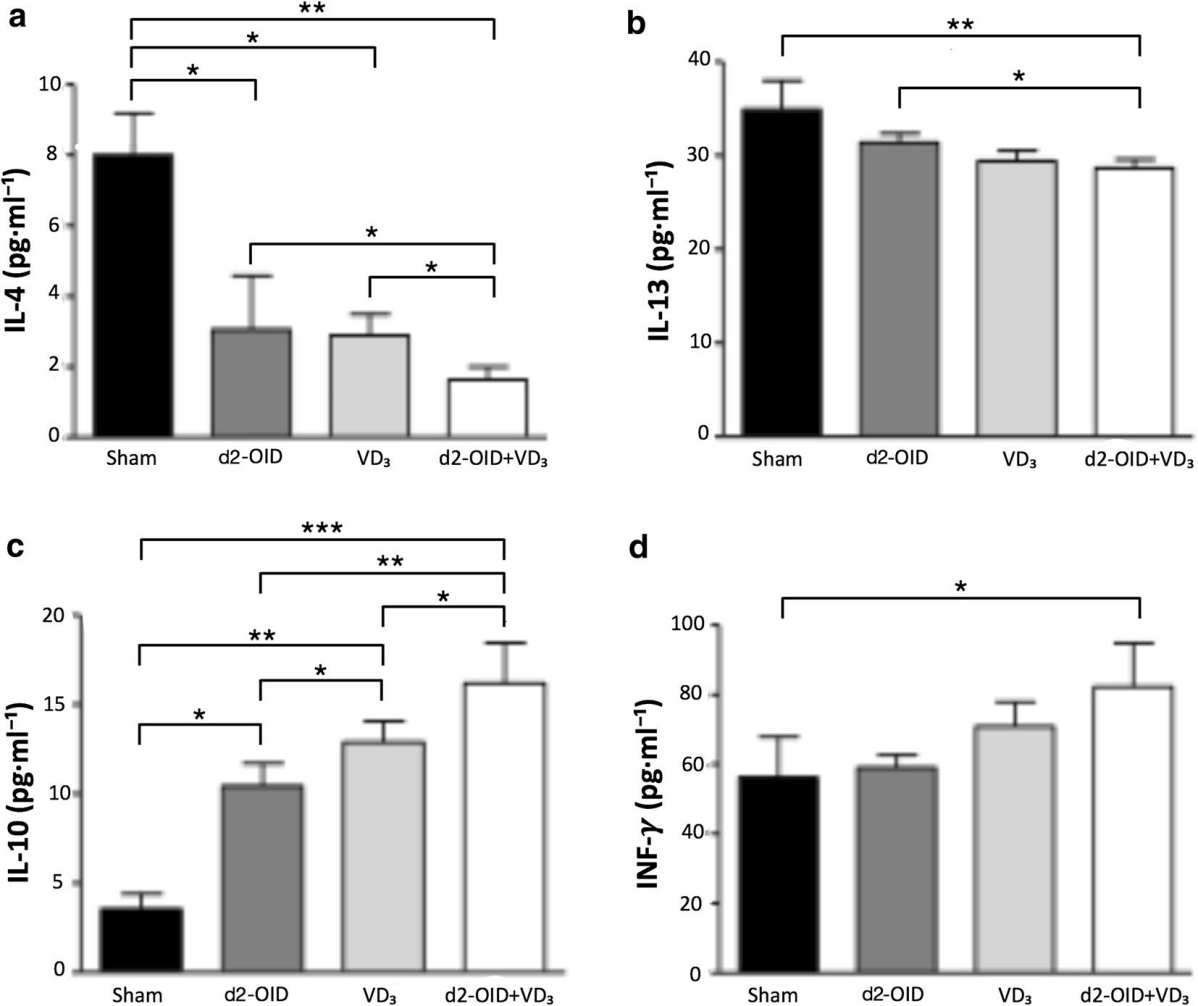
Treatment-induced modulation of cytokines’ levels in BALF. The levels of IL-4, IL-13, IL-10, INF-γ in BALF were measured by ELISA. The conventional low dose IT with d2-OID or the sole VD_3_ administration induced decrease of IL-4 (**a**) and increase in IL-10 (**c**) and no change in the level of IL-13 and IFN-γ. Only the association d2-OID + VD_3_ produced the highest modification of these cytokines, well-matched with the resolution of the allergic inflammation, induction of tolerance and immune deviation: in fact, IL-4 (**a**) and IL-13 (**b**), were significantly lower and IL-10 (**c**) and IFN-γ (**d**) were significantly higher, compared with all the other groups. Values are expressed as mean ± SD (n = 12). *p < 0.05, **p < 0.01, ***p < 0.001

The level of IL-4 was significantly lower after d2-OID (p < 0.05 vs sham) or VD_3_ (p < 0.05 vs sham) treatment, and even less in the mice who received the combined administration d2-OID + VD_3_ (p < 0.01 vs sham; p < 0.05 vs d2-OID and VD_3_) (Fig. 3a). All treatments seem to produce a slight decrease in IL-13 level, but only the d2-OID + VD_3_ treatment was associated with a significant modification (p < 0.01 vs sham and p < 0.05 vs d2-OID treatments) (Fig. 3b). A significant increase of IL-10 was observed for all treatments (showing the trend: sham < d2-OID < VD_3_ < d2-OID + VD_3_); the latter produced the highest and most significant variation compared with each of the other three treatments (p < 0.001 vs sham, p < 0.01 vs d2-OID and p < 0.5 vs VD_3_) (Fig. 3c). INF-γ increased in the groups of mice VD_3_ and d2-OID + VD_3_, but such change reached the significance (p = 0.05) only in the group of mice that received the combined one (Fig. 3d). TNF-α significantly decreased for the all groups of mice, but the change reached the highest significance for the d2-OID + VD_3_ group (p < 0.01) (not shown). IL-6 level showed a general trend of reduction, that was significant only for d2-OID-VD_3_-treated mice (p < 0.05) (not shown). For better understanding, all statistical analysis data are summarized in Table 1.

**Table 1:** Significance of variations of the parameters amongst the experimental groups

Vs	D2-OID	VD3	D2-OID + VD3
D2-specific IgG2a (serum)			
Sham	n.s.	*	**
D2-OID		*	**
VD3			*
IL-4 (BALF/lung homogenate)			
Sham	*	*	**
D2-OID		n.s.	*
VD3			*
IL-13 (BALF/lung homogenate)			
Sham	n.s	n.s.	**
D2-OID		n.s.	*
VD3			n.s.
IL-10 (BALF/lung homogenate)			
Sham	*	**	***
D2-OID		*	**
VD3			*
IFN-γ (BALF/lung homogenate)			
Sham	n.s.	n.s.	*
D2-OID		n.s.	n.s.
VD3			n.s.
TNF-α (BALF/lung homogenate)			
Sham	*	*	**
D2-OID		n.s.	n.s.
VD3			n.s.
IL-6 (BALF/lung homogenate)			
Sham	n.s.	n.s.	*
D2-OID		n.s.	n.s.
VD3			n.s.
Treg (FoxP3^+^ CD25^+^ % of CD4^+^, spleen)			
Sham	*	**	**
D2-OID		*	*
VD3			n.s
Treg (FoxP3:CD3, lung)			
Sham	*	*	***
D2-OID		n.s.	**
VD3			**
Eosinophils (lung)			
sham	n.s.	P = 0.02	(0.01)**
D2-OID		(0.05)*	P = 0.02
VD3			P = 0.051

*p < 0.05, **p < 0.01, ***p < 0.001

### Determination of T regulatory cells

**Fig. 4:**
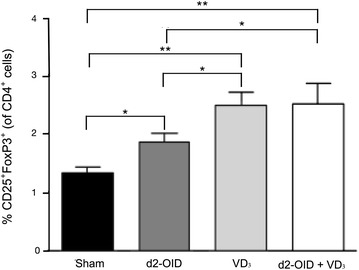
Frequency of Tregs in spleen cells. Flow cytometry data showing the percentage of FoxP3^+^CD25^+^ cells on gated CD4^+^ cells of total spleen cells. The d2-OID treatment produced an increase of this subpopulation of regulatory cells (p < 0.05 vs sham) but not as high as for the other groups of treatment. Either VD_3_ or d2-OID administration induced the highest increase in FoxP3^+^CD25^+^ cells, compared with sham (p < 0.01, for both groups) or d2-OID (p < 0.05, for both groups). Values are expressed as mean ± SD (n = 12). *p < 0.05 and **p < 0.01

In the spleen, the frequency of CD4^+^CD25^+^FoxP3^+^ cells assessed by FACS was significantly higher in all groups of treatment, compared with the sham group, although at different extent: the sole d2-OID treatment induced a limited increase Tregs (p < 0.05 vs sham), whereas the VD_3_ and d2-OID + VD_3_ groups showed a more evident and comparable change (p < 0.01 vs sham and p < 0.05 vs d2-OID, for both) (Fig. 4).

**Fig. 5:**
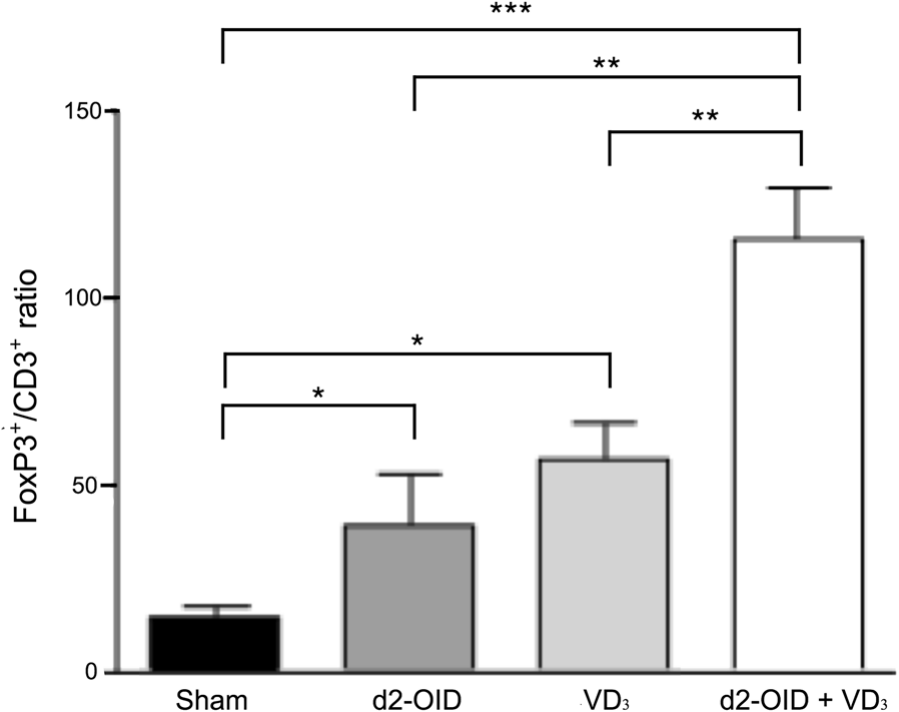
Abundance of T regulatory cells in lung tissue. A significant comparable increase in FoxP3^+^:CD3^+^ cells ratio was induced by d2-OID and VD_3_ treatments (p < 0.05 vs sham); the combination treatment d2-OID + VD_3_ was even more efficacious in inducing such an increase (p < 0.001 vs sham, p < 0.01 vs d2-OID and VD_3_ treatments) Values are expressed as mean ± SD (n = 7). ***p < 0.001 **p < 0.01. *p < 0.05

In the lung, the frequency of CD3^+^ T cells expressing the T regulatory cell marker FoxP3^+^ assessed by histochemical analysis showed Foxp3^+^/CD3^+^ cells ratio is significantly higher in mice treated with d2-OID + VD_3_, compared to all groups: by 84 % compared with sham treated mice (p < 0.001), by 55 % compared with d2-OID-treated mice (p < 0.01) and by 34 % compared with VD_3_-treated mice (p < 0.01). Increase in the abundance of Tregs was also associated with the other two treatments, although these differences were found less significant (p < 0.05 vs sham, for both) (Fig. 5).

### BALF eosinophilia

**Fig. 6:**
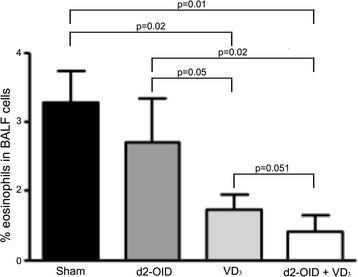
Eosinophils abundance in BALF. The frequency of eosinophils cells in total BALF cells showed a trend in diminution following d2-OID treatment, but it was significantly decreased only with the treatments VD_3_ (p = 0.02 vs sham and p = 0.05 vs d2-OID) and d2-OID + VD_3_ (p = 0.01 compared with sham and p = 0.02 vs d2-OID). The d2-OID + VD_3_ induced a further decrease in eosinophils at the limit of significance, compared to VD_3_ (p = 0.051). Values are expressed as mean ± SD (n = 7)

Sensitization and challenge with Der p 2 significantly increased the frequency of BALF eosinophils in the sham-treated group compared with the naive group (not shown). The d2-OID treatment alone reduced (not significantly) the eosinophils abundance, whereas these inflammatory cells resulted significantly reduced in the VD_3_ alone group (p = 0.02 vs sham) and in mice treated by the combination d2-OID + VD_3_ (p = 0.01 vs sham). Moreover, the d2-OID + VD_3_ treatment was associated with the highest decrease, although at the limit of significance, compared with the d2-OID treatment (p = 0.02) and the VD3 treatment (p = 0.051) (Fig. 6).

## Discussion

In our study, we investigated whether VD_3_, as an adjuvant for AIT, would be efficacious when administered in combination with a low dose of antigen which, if taken alone, had no power to restore a non-pathological immune response to the native allergenic protein.

We found that VD_3_ is clearly able to enhance the effects of AIT with allergoid in a mouse model of allergic asthma to dermatophagoides. The main finding of the study was the modulation of the local allergic inflammation in the lung obtained by the VD_3_-adjuvated allergoid AIT. In particular, it induced an increase in Tregs in the lung and a decrease in BALF eosinophils along with a Th1/Th2 shift: increase of d2-specific IgG2a antibodies, interferon-γ and IL-10 parallel to a reduction of IL-4 and IL-13. These changes were statistically significant when compared to sham and d2-OID treated mice, showing the effectiveness of VD_3_ as adjuvant for AIT in restoring the immune tolerance against the sensitizing allergen. Actually in literature there are demonstrations that the biologically active form of VD_3_ is able of modulating the innate and adaptive immune response [7–9]. VD_3_ was shown to induce a non-specific up-regulation of IL-10 and TGF-β [14] and of Tregs also in an experimental autoimmune disease model [20].

Also in our experiments VD_3_ was able to induce an increase in Tregs, particularly in the spleen where there was no significant differences in the increase in regulatory T lymphocytes between VD_3_ and adjuvated allergoid vaccine treated mice, in both cases significantly higher than sham and allergoid treated mice. By contrast, in the lung Tregs frequency increases significantly only in d2-OID + VD_3_ treated mice (Table 1). It seems that allergoid + VD_3_ co-administration confers specificity to the VD_3_ adjuvant action: the Tregs abundance was similar for the two groups treated with VD_3_ or allergoid + VD_3_ in the periphery, whereas, only the latter treatment produced the highest accumulation of Tregs exactly in the lung. This could be obtained through a homing driven by chemokine receptors expressed on their cell surface [26]. Moreover, the allergen-specific TcR of Tregs might have played a role in their localization in the inflamed lung as the inhaled allergen could be presented by endothelial and parenchymal cells allowing trans-endothelial migration and tissue retention [27]. In this way the AIT approach is the only one able to increment locally the Treg cell population and represents the best choice for allergy management. Other strategies for the development of anti-allergic compounds are focusing on the inhibition of the migration of inflammatory cells in the lung, acting on the CCR-ligand interaction [28]. However, these kinds of approaches might have important side effects since they block homing receptors that are non specific for a certain cell type but are shared amongst different ones. Therefore, we believe that the strategy of incrementing the allergen-specific Tregs population into the lung of allergic subjects might be more effective in the extinction of allergic inflammation with reduced risk of detrimental outcomes.

Furthermore, a reduction of the eosinophils accumulation was found in the lungs of both VD_3_ alone and the VD_3_-adjuvated AIT treated mice, significantly higher than allergoid-treated mice. Again, VD_3_ + allergoid association appeared to be more efficacious than VD_3_ alone. Although these data may be affected by bias due to semiquantitative evaluation, it is plausible that the effects of the low dose of allergoid used in our experiments are misted up by the effects of the VD_3_ and that a further reduction of eosinophils might be achieved with a higher dose of allergoid. Coherently with the highest accumulation of FoxP3^+^ T cells in the lung of VD_3_ adjuvated AIT treated mice we observed a significant decrease of Th2 and regulatory citokines in BALF.

Moreover, it is expected that the VD_3_ + d2-OID association may have a longer lasting effect compared to other treatments, as suggested by the induction of the highest levels of IFN-γ in the lung. In fact, the induction of this cytokine, a typical product of Th1 cells, confirms the occurrence of the so called Th2/Th1 shift needed for a successful AIT [1]. A similar plan to obtain long-term effect of allergen-specific immunotherapy in a murine model of type I allergy (towards OVA) has been described by Heine and colleagues [15]. Differently, they used as comparison terms deficient VD_3_ dose and, as therapy, the precursor of VD_3_, to mimic the condition characterizing people living at certain latitudes, and to avoid ipercalcemia-associated toxicity. They conclude that VD_3_ correction helps the efficacy of AIT [15].

In our settings, we mimic the other condition of a normal level of serum VD_3_, essential for the correct ongoing of a plethora of immunological and biological processes, and the possible effect of a non-toxic supplementation on AIT.

Future developments of the present study could include the demonstration that the combined administration of VD_3_ and allergoid in an AIT setting can act in a specific manner and that the allergoid ensures the tolerogenic/inhibitory action limitedly to the Th2 allergen-specific cells and that such specificity promotes the accrual of Tregs at the inflammation sites. The specificity of the observed Tregs-mediated response in vivo and the effective suppression of the airway inflammation will be assessed with functional studies in vitro (inhibition studies) and in vivo (pulmonary function).

## Conclusions

The concept to use vitamin D_3_ as an adjuvant immunomodulant factor in allergy treatment has been explored in this study. The most relevant finding regards the accumulation of Tregs in the lung associated with the treatment of sensitized and airway-challenged mice by immunotherapy based on 2d-OID + VD_3_ administration. This effect appears to be allergen-driven and allergen-specific. This is a promising result for improving the current AIT. In particular, it is envisaged that the covalent linking of VD_3_, directly or indirectly, to the allergoid would allow to achieve highest local concentration of both compounds [21]. Practically, the addition of VD_3_ to a conventional AIT protocol would allow the reduction of allergoid dose needed and therefore, the production costs. Moreover, this study shows that important immunomodulatory effects can be achieved by the oral administration. This modality of treatment might favour the management of the therapy by the patients and their adherence.

This represents a further and particularly interesting development of the described approach as it might enhance the efficacy of the observed immune modulation. However, first, confirmative experiments on the specificity and functional role of the Treg population is needed.

## Additional file

10.1186/s12948-016-0044-1 Additional material and figures.

## Abbreviations

AIT: allergen-specific immunotheraphy
BALB/c: inbred strain of mouse
BALF: bronchoalveolar lavage fluid
d2-OID: chemically-modified monomeric allergoid of Der p 2
DC: dendritic cell
HDM: house dust mite
ELISA: enzyme-linked immunosorbed assay
Eo: eosinophil
Ig: immunoglobulin
IL-10: interleukin-10
MuSC: mucous secretory cells
OVA: ovalbumin
GF-β: transforming growth factor β
PBMC: peripheral blood mononuclear cells
RT: room temperature
Th2: T-helper cell type 2
Tregs: T regulatory cells
VD_3_: vitamin D_3_
