# Supplementary material for: Vitamin D3 improves the effects of low dose Der p 2 allergoid treatment in Der p 2 sensitized BALB/c mice
Source: Clin Mol Allergy. 2016 Aug 5;14:7. doi: 10.1186/s12948-016-0044-1 (PMC4975903; doi:10.1186/s12948-016-0044-1)
Supplement: Supplementary file 1 — 10.1186/s12948-016-0044-1 Additional material and figures. [file 12948_2016_44_MOESM1_ESM.docx]

PETRARCA C ET AL. ADDITIONAL MATERIAL TO MANUSCRIPT ENTITLED: “Vitamin D3 improves the effects of low dose Der p 2 allergoid treatment in Der p 2 sensitized Balb/c mice”

SUPPLEMENTARY MATERIAL AND METHODS

Production and purification of rDer p 2 in Escherichia coli

Der p 2-encoding cDNA flanked by a sequence coding for six histidines was cloned in a prokaryotic vector (pEt 3c, Stratagene, La Jolla, CA) and expressed in Escherichia coli cells (Origami B, Novagen, Merck Biosciences, Darmstadt, Germany) according to manufacturer’s protocols (Figure S1). Clones obtained from single bacterial colonies were sequenced according to Sanger to verify the correct cDNA sequence. The cells were harvested by centrifugation, resuspended in a 50 mM NaH2PO4, 300 mM NaCl buffer, pH 8, and lysed by sonication. The recombinant proteins were separated by centrifugation. The pellet containing an insoluble protein aggregate was resuspended in 100 mM NaH_2_PO_4_, 10 mM Tris-HCl, 8 M urea (pH 8) (denaturing buffer) and stirred for 60 min. The solubilized recombinant proteins were separated from insoluble debris by centrifugation and purified by affinity chromatography under denaturing conditions using nickel-nitrilotriacetic acid (Ni-NTA) agarose columns (Qiagen SpA, Milan, Italy). Immobilized His-tagged proteins were refolded on the Ni-NTA columns, using a linear 6 M-0 M urea gradient in 50 mM NaH_2_PO_4_, pH 8 at RT over a period of 1.5 h. After renaturation, purified proteins can be eluted by the addition of 250 mM imidazole and dialysed for 16 hours at 4°C in 0.68% NaCl, 0.275% NaHCO_3_ solution and stored at -20 °C or submitted to chemical modification. After purification, rDer p 2 was electrophoresed on a denaturing 4-12% polyacrilamide precast Nupage Bis-Tris gel according to manufacturer’s instructions (Novex, Life Technologies, Carlsbad, California), migrating as a single band and displaying more than 98% purity. Recombinant Der p 2 was recognized in ELISA assays by IgE antibodies from allergic patients’ sera (Figure S2) and by monoclonal antibody raised against natural Der p 2 (data not shown).

Chemical modification with KCNO of rDer p 2

Purified rDer p 2 was submitted to chemical modification as previously described [1]. Briefly, solid sodium tetraborate and potassium cyanate were added to samples at final concentrations of 0.1 M and 0.5 M, respectively. After 20 hours at 40°C, salts were eliminated by gel-filtration on G-25 column (PD-10 Desalting Columns, GE Healthcare Bio-Sciences AB, Uppsala, Sweden) and samples were characterized by ELISA as IgE reactivity in comparison with the corresponding native counterpart.

Dermatophagoides pteronyssinus extract

*Dermatophagoides pteronyssinus* (HDM) bodies were extracted by magnetic stirring (24 h at 4°C) in 0.15 phosphate buffer, pH 7.2 at 5% w/v. The extract was clarified by centrifugation at 16,300 g and dialyzed in 5 mM (NH_4_)HCO_3_. Determination of protein concentration of purified rDer p 2 or HDM extract was performed according to Bradford [2] using the commercial BioRad Protein Assay Dye Reagent (Bio Rad, Milan, Italy). Endotoxin removal was obtained by processing the purified proteins or the extract in the Amicon Ultra-15 50K centrifugal filter device (Merck Millipore Ltd, Darmstadt, Germany). The content of lipopolysaccharide in the ultrafiltrated rDer p 2 or HDM preparations was determined by the Limulus amebocyte lysate test (VWR International PBI Srl, Milano, Italy) according to the instructions of the manufacturer.

**Assessing the low AIT dose of allergoid**

In order to determine the low (suboptimal) dose for AIT, three goups of Der p2-sensitized mice received immunotherapy with on three different doses of d2-OID (0.1, 5 and 10 µg) of by orogastric gavage, five times a week for 3 weeks. Not sensitized, healthy mice (naïve) and only sensitized mice (sham) were used as control groups. In addition, as a control group for the antigen specificity of the airway inflammation, mice were immunized with 1 µg i.p. injections of OVA prior to challenge the lungs with HDM extract aerosol. Specific-IgE serum level and cellular infiltration in the lungs were analyzed 48 hours after the last HDM airway challenge.

# Results

Preliminary Experiment: determination of the suboptimal d2-OID dose for AIT

Der p 2-specific IgE levels in serum

Specific serum IgE was significantly increased (p<0.001) in the sham group compared to the naive group, indicating that the allergic sensitization had been successfully evoked. Also the treatment groups showed significant levels of IgE in comparison with the naive group; however, compared with the sham treated group. an inverse d2-OID dose- IgE titre was observed. In fact, 10 µg of d2-OID produced the highest, tough not complete, diminution of Der p 2-specific IgE by 61% (p<0.001); also 5 µg of d2-OID significantly suppressed these Ig, although at a lesser extent (18 %, p<0.05); the lowest dose of d2-OID (0.1 µg) was associated to a negligible reduction of anti-Der p 2 IgE (Figure S3). In the control mice used to verify the specificity of the local immune response to the aerosolized allergen (sensitized to the non-related antigen Ovalbumin and HDM-challenged), no IgE anti-Der p 2 were detected (data not shown).

Lung eosinophilia

To determine the efficacy of d2-OID on lung eosinophilia, the inflammatory cells in the lung tissue were evaluated 48 h after the last aerosol challenge with HDM extract, comparing each of the three treatment groups with the sham group (Figure S4). 10 µg of d2-OID effectively suppressed the eosinophilia; 5 µg of d2-OID partially suppressed the eosinophils accumulation, but this reduction did not reach the level of significance (Figure S5); no reduction was found in mice treated with the lowest dose of 0.1 µg of d2-OID. (data not shown). No signs of inflammation were detected in the lungs from mice sensitized with OVA and exposed to HDM aerosol, demonstrating that the airway response was dependent on specific sensitization against Der p 2 (data not shown). Therefore, to assess the role of VD3 in AIT the suboptimal dose of 3 µg per mouse daily was chosen to ensure a lower decrease of anti-Der p 2-IgE respect to 5 µg.

**Figure Legends**

Figure S1. Production and characterization of recombinant Der p2.

1. Map of vector pET-3c. (B) Western blot analysis of total extract from rDer p2-producing E. coli.

**A**

B
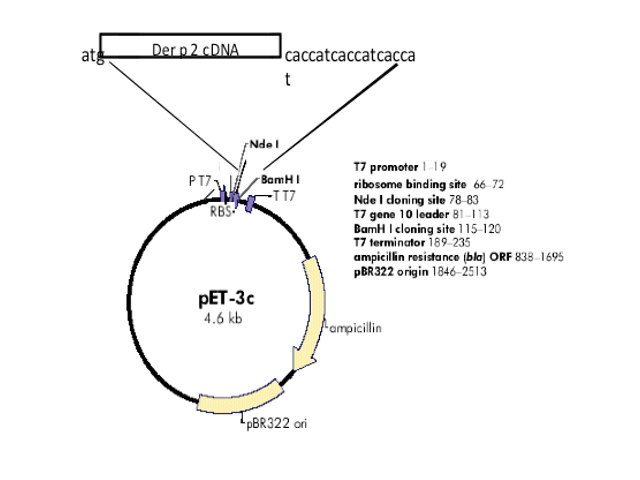


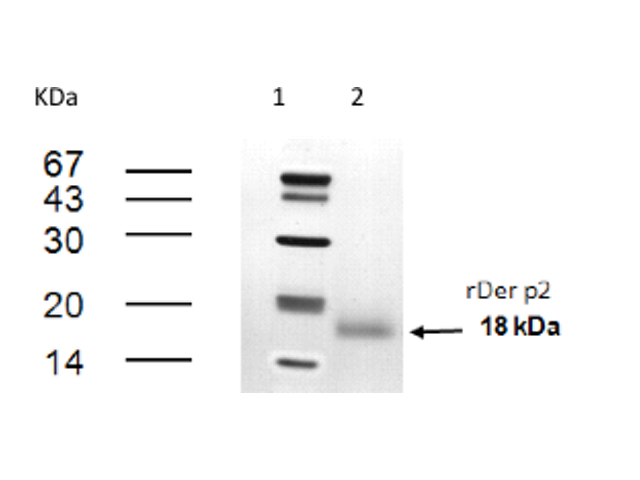


Figure S2. Immunoreactivity of recombinant Der p2.

Comparison of human IgE antibody binding to native and chemically modified recombinant Der p 2 (rDer p 2). Immunoreactivity of a pool of sera from ten mites-allergic patients was analyzed by ELISA assay. Ig E reactivity was tested as a function of different dilutions of pooled sera.


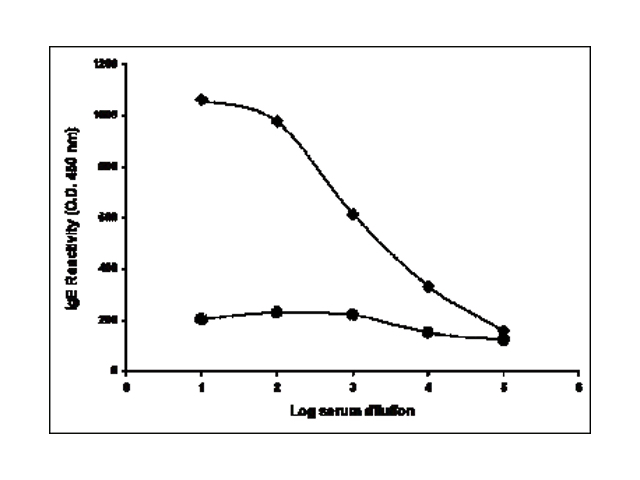


**Figure S3.** Experimental design


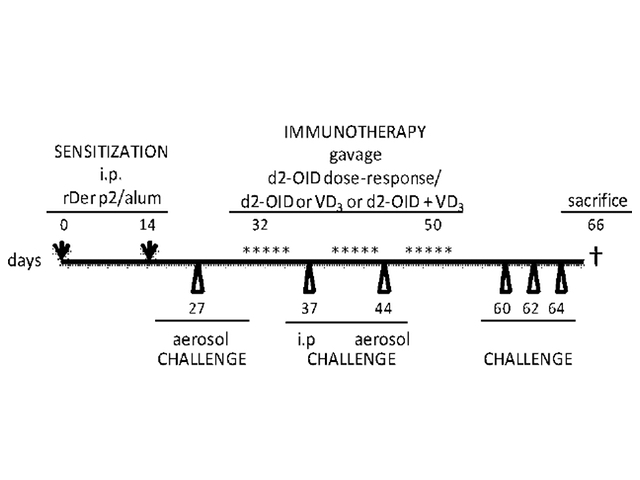


Figure S4. Analysis of Der p2-specific IgE response to determine suboptimal d2-OID dose. Sensitized mice are treated with three different doses of allergoid-IT: 0.1 µg, 5 µg and 10 µg. Levels of Der p2-specific IgE were analyzed in serum of all groups two days after HDM extract aerosol challenge. Values are expressed as mean ± SD (*n* = 4). $$$ *p* < 0.001 compared with naïve mice; * *p* < 0.05 and *** *p* < 0.01 compared with Sham-treated mice.


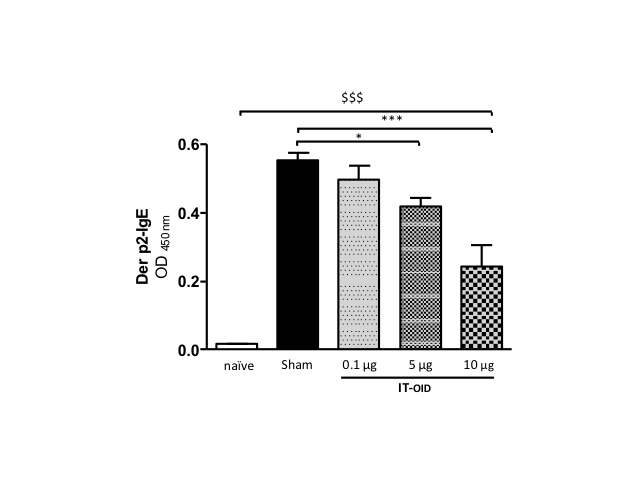


Figure S5. Determination of suboptimal IT-OID dose.

Lung histological examination by H&E. Challenge with HDM extract in naïve mice caused no airway inflammation. (naïve/challenged mouse); Sham are Der p2 sensitized/HDM challenged mice; mouse treated with 5 ug d2-OID.


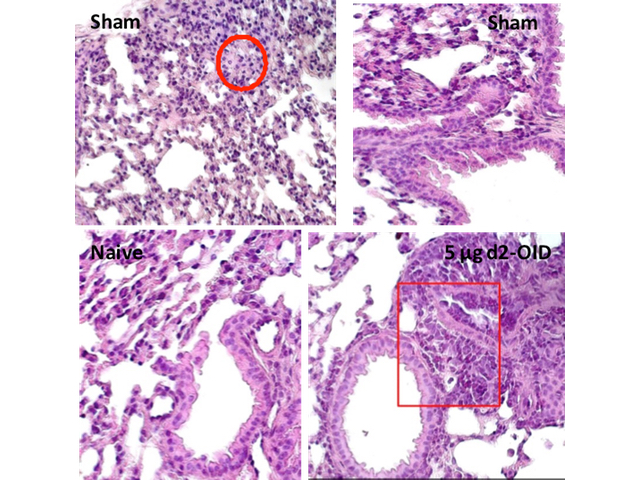


**References**

1. Mistrello G, Brenna O, Roncarolo D, Zanoni D, Gentili M, Falagiani P. Monomeric chemically modified allergens: immunologic and physicochemical characterization. Allergy. 1996;51: 8–15.

2. Bradford MM. A rapid and sensitive method for the quantitation of microgram quantities of protein utilizing the principle of protein-dye binding. Anal Biochem. 1976;72: 248–254. doi:10.1016/0003-2697(76)90527-3
